# Prediction of high anti-angiogenic activity peptides in silico using a generalized linear model and feature selection

**DOI:** 10.1038/s41598-018-33911-z

**Published:** 2018-10-24

**Authors:** Jose Liñares Blanco, Ana B. Porto-Pazos, Alejandro Pazos, Carlos Fernandez-Lozano

**Affiliations:** 1 Department of Computer Science, Faculty of Computer Science, University of A Coruña, A Coruña, 15071 Spain; 2 Instituto de Investigación Biomédica de A Coruña (INIBIC). Complexo Hospitalario Universitario de A Coruña, A Coruña, Spain

## Abstract

Screening and *in silico* modeling are critical activities for the reduction of experimental costs. They also speed up research notably and strengthen the theoretical framework, thus allowing researchers to numerically quantify the importance of a particular subset of information. For example, in fields such as cancer and other highly prevalent diseases, having a reliable prediction method is crucial. The objective of this paper is to classify peptide sequences according to their anti-angiogenic activity to understand the underlying principles via machine learning. First, the peptide sequences were converted into three types of numerical molecular descriptors based on the amino acid composition. We performed different experiments with the descriptors and merged them to obtain baseline results for the performance of the models, particularly of each molecular descriptor subset. A feature selection process was applied to reduce the dimensionality of the problem and remove noisy features – which are highly present in biological problems. After a robust machine learning experimental design under equal conditions (nested resampling, cross-validation, hyperparameter tuning and different runs), we statistically and significantly outperformed the best previously published anti-angiogenic model with a generalized linear model via coordinate descent (glmnet), achieving a mean AUC value greater than 0.96 and with an accuracy of 0.86 with 200 molecular descriptors, mixed from the three groups. A final analysis with the top-40 discriminative anti-angiogenic activity peptides is presented along with a discussion of the feature selection process and the individual importance of each molecular descriptors According to our findings, anti-angiogenic activity peptides are strongly associated with amino acid sequences SP, LSL, PF, DIT, PC, GH, RQ, QD, TC, SC, AS, CLD, ST, MF, GRE, IQ, CQ and HG.

## Introduction

The angiogenesis process consists of the growth and development of new blood vessels from existing ones. The continuous interaction between endothelial cells and the cellular environment that surrounds them is fundamental for this process to occur. Under normal conditions, there is constant regulation between inhibitory and promoter molecules in this process, generating a correct vascularization of tissues^1^.

The study of this field has grown enormously in recent years due to the discovery of effective anti-angiogenic therapies in numerous fields, including dermatology^2^, ophthalmology^3^, vascular diseases^4^ and oncology^5^.

Cancer research is the area in which most studies are being conducted due to the increased incidence of this disease across the population. Recent data from the National Institute of Health (NIH) indicate that between 2012 and 2030, the incidence of cancer is expected to rise by 50%, from 14 to 21 million patients a year. As to the number of deaths, an increase of 60% is expected, from 8 to 13 million deaths a year.

Previous studies have shown that cancer cells induce the growth of the blood vessels around them, providing them with nutrients and molecules vital for their development^6^. In addition, many cancer types have been reported as being dependent on the process of angiogenesis and respond well to anti-angiogenic therapies^7^. The promising results obtained by researchers and the FDA approval of drugs that inhibit this process as a treatment for various types of cancer have led to the development of a therapy focused on the inhibition of cellular angiogenesis from multiple small peptides, each with a specific target on different metabolic pathways^1^.

A peptide is constituted by the union of amino acids by peptide bonds. The main difference from proteins is its size and structure. Peptides are smaller (between 2 and 50 amino acids) and do not have complex, tertiary or quaternary structures. These characteristics have a series of advantages when peptides are used as therapeutic agents. On the one hand, they are small, organic molecules with a very low level of toxicity. They present, on the other hand, great specificity when joining with other molecules, which facilitates a targeted therapy for a variety of tissues. In addition, they can be designed *in vitro*^1^. Given the simple characteristics of these molecules, it is easy to see that an amino acid sequence will be crucial for the presence of anti-angiogenic function. Previous studies reported that the presence of residues such as Cys, Pro or Ser is related to this activity, while residues such as Ala, Asp or Ile have the opposite functionality^8^.

A metabolic pathway is a molecular process involving various gene products with the aim of performing a specific function. The interaction among the various proteins can be direct (phosphorylation, acetylation, etc.) or indirect (via second messengers such as cAMP). The coordination of all molecules is crucial for the correct functioning of the route. This is why the inhibition of a molecule can be considered as a therapeutic target to stop a certain activity or molecular pathway.

Because of the high cost and low speed of the experimental techniques used to evaluate the presence of any peptide activity, researchers increasingly rely on *in silico* experiments for the a priori prediction of the possible activities of each peptide. Previously, these methods *in silico* were based solely on searching in the scientific literature and public databases–for example, the search for protein domains^9^, the genomic and proteomic study later contrasted by the null hypothesis theory^10^ or the sequence homology^11^. In this way, a theoretical framework was presented prior to evaluation by experimental techniques, owing largely to massive projects such as the Human Genome Project, thus achieving great savings in economic, time and human resources.

After the implementation of more powerful statistical and computational techniques in the biomedical field and with the drastic reduction of costs in the acquisition of hardware, the theoretical framework was strengthened, and machine learning (ML) algorithms, among others, began to be used. Thus, with the use of classification algorithms, models with high performances were obtained^8,12–14^, achieving great success in the classification of peptide activities^15^, cell-penetrating peptides^16^ or anti-cancer peptides^17^.

The classification model reported in the present paper represents a quantitative structure activity relationship (QSAR) between the protein amino-acid composition and the biological function. Previous studies on other protein functions focused on anti-oxidant^18^, transporter^19^, cell-penetrating^20^, anti-viral^21^, enzyme regulator^13^, cell death-related^22^, cancer-related^23,24^, microbiome-related^25^ or signaling^12^ proteins.

Regarding anti-angiogenic activity, most studies were based only on the experimental part^1,26–28^, which greatly increased the cost and time spent in the characterization process. Although there are also studies that have implemented algorithms based on ML^8^, the highest prediction performance value is closest to 0.81 in accuracy, and this study did not report any combined measure to control overfitting or type I and II errors.

**Figure 1:**
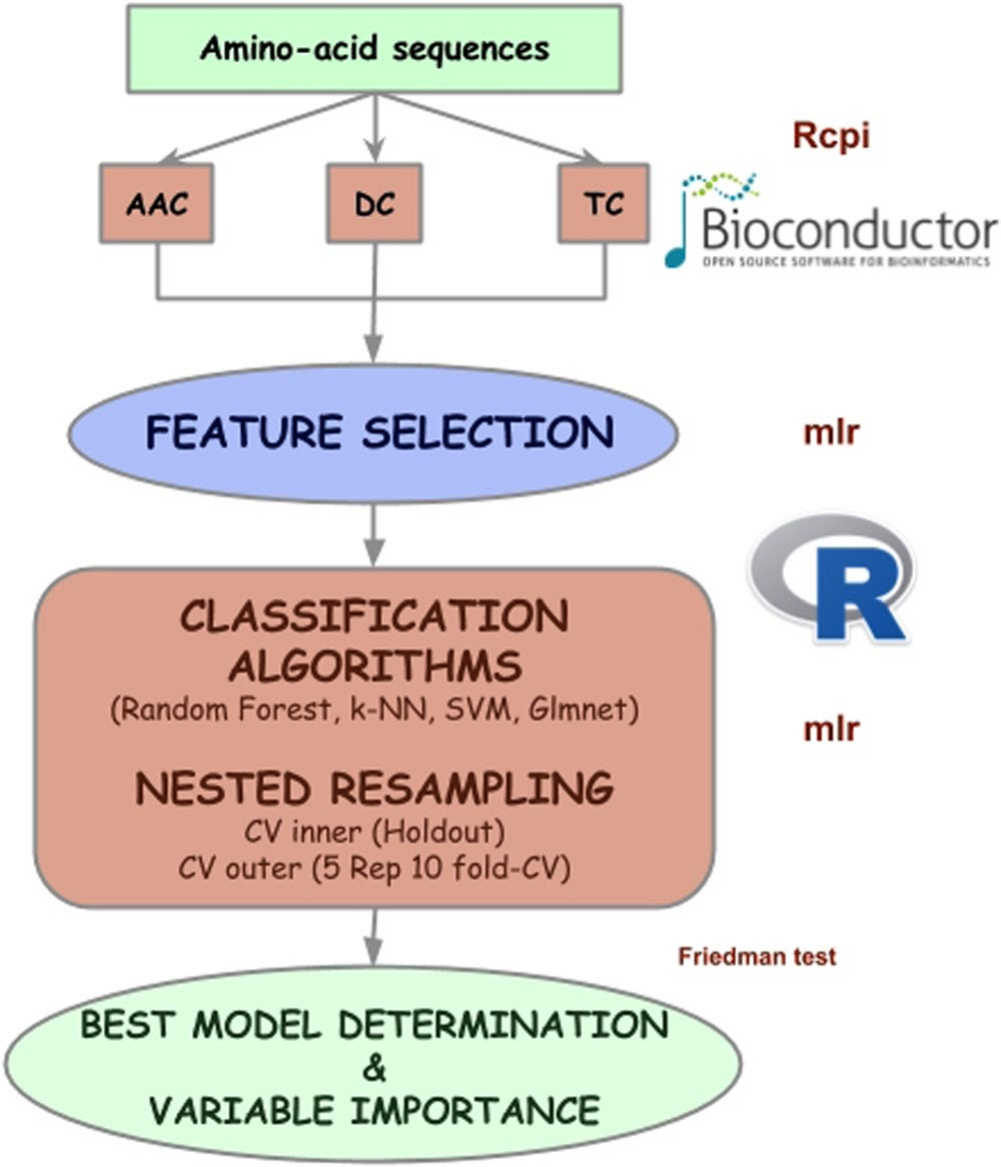
Flowchart of this study. The authors thank Bioconductor and R (https://www.r-project.org/logo/) for the provision of the logos under CC-BY and CC-BY-SA open access licenses.

Due to the need to strengthen the theoretical framework in the prediction of peptides with anti-angiogenic activity, our aim is to obtain the molecular descriptors, select the best variables within the descriptors regardless of the descriptor and look for the machine learning algorithms that better convey the underlying knowledge in the data. With the combination of these different stages, as shown in Fig. 1 new information on anti-angiogenic activity will be obtained, and an effective predictive model will be presented to ensure that one specific peptide will be a potential candidate for evaluation through experimental techniques.

Once the state of the art and the present study have been introduced, we move on to discuss the structure of this paper. First, the results are divided into several subsections: baseline algorithms without feature selection, feature selection and best model determination. This is followed by a discussion and the conclusions of this study. Finally, the materials and methods section contains a brief introduction to the dataset, molecular descriptors, machine learning, feature selection and experimental design used in this work.

## Results

Previous studies have shown that anti-angiogenic peptides have common functionality, structure and composition^1,10^. Regarding the structure, the vast majority of folds are anti-parallel beta sheets and contain a relatively high incidence of hydrophobic and cationic residues^10^. Furthermore, it has been shown that such peptides are more prone to have certain residues and amino acid sequences in their composition, although this feature is not completely defined. Alignment analysis has not indicated significant sequential commonalities among the peptides^10^. Therefore, the present study aims to elucidate fundamental aspects in the study of the aminoacidic composition of these peptides.

### Baseline algorithms without feature selection

**Figure 2:**
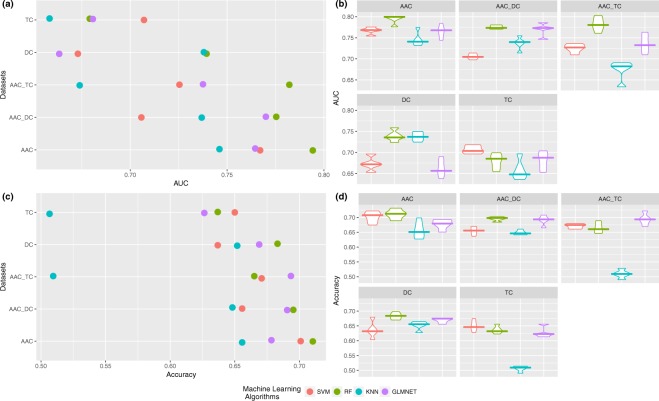
Results obtained with the original datasets AAC, TC and DC and their combination. (**a**) Summary of the performance of the four algorithms (AUC), (**b**) boxplot of the behavior of each model across experiments (AUC), (**c**) summary of the performance of the four algorithms (accuracy), and (**d**) boxplot of the behavior of each model across experiments (accuracy).

We used four different machine learning algorithms: RF, SVM, k-NN and glmnet. Initially, we considered the three original datasets (AAC, TC, DC) and merged them. As shown in Fig. 2a, the most informative dataset is AAC, and the merging of the datasets (AAC_TC and AAC_DC) significantly improves the performance of the models (DC and TC) while slightly reducing the deviation of the results, as shown in Fig. 2b.

Results do not improve those published before in the literature (0.809 in accuracy) using a SVM and NT15 terminus dataset (contain first fifteen residues from the N-terminal region of the peptide sequence), as shown in Fig. 2c, but to reduce the noise in the datasets, an FS approach should be applied. Figures in this paper were built using the ggplot2 package^29^.

**Figure 3:**
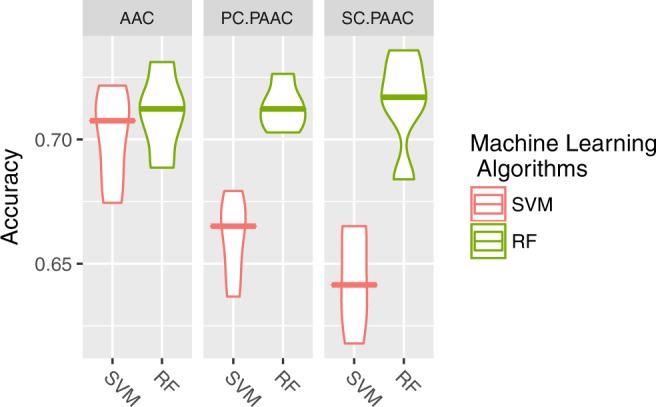
Results obtained with the RF and SVM algorithms using AAC and the novel parallel correlation pseudo-amino-acid composition and series correlation pseudo-amino-acid composition.

In conclusion, in terms of both AUC and accuracy, the best result was obtained by the RF algorithm trained with the AAC dataset, as shown in Fig. 2a,c. The TC and DC datasets, generally, achieved lower performance with all algorithms than AAC and the combination of them. In light of these results, we considered two complementary AAC descriptors: parallel correlation pseudo-amino-acid composition (PC-PseAAC) and series correlation pseudo-amino-acid composition (SC-PseAAC)^30^. As shown in Fig. 3 (violin plot), the two better-performing models in terms of accuracy with the AAC dataset, RF and SVM (Fig. 2c) had opposite behaviors. On the one hand, RF (best model) achieved a comparable result with a similar median value for PC-PseAAC but with outliers for SC-PseAAC in the lower part of the plot; this seems to indicate that it is less stable than AAC^31,32^. However, we found that SVM achieved significantly poorer results with a clear decreasing trend in the performance for datasets PC-PseAAC and SC-PseAAC; this is consistent with other works published in the literature^33^. Furthermore, as mentioned before, the aim of this work is to find the aminoacidic composition capable of biologically explaining the differences between anti-angiogenic and non-anti-angiogenic peptides. Due to this instability and to the fact that biologically, AAC is easy to understand, explain and validate by biological lab methods, we decided to use the original AAC dataset for the feature selection experiments.

More precisely, on a closer inspection of the boxplots in Fig. 2b,d, all models show a high variance in their results across experiments, especially the models trained with the descriptors that present a greater number of variables (DC and TC).

### Feature selection

At this point, we performed an FS approach to reduce the noise in the three original datasets (AAC, DC, TC) and merged them. We ranked the features for each dataset and explored the sizes of different subsets, (5, 10 and 15) for AAC, (25, 50, 75 and 100) for DC, (75, 100, 125 and 150) for TC and (50, 100, 150 and 200) for the union of the three.

**Figure 4:**
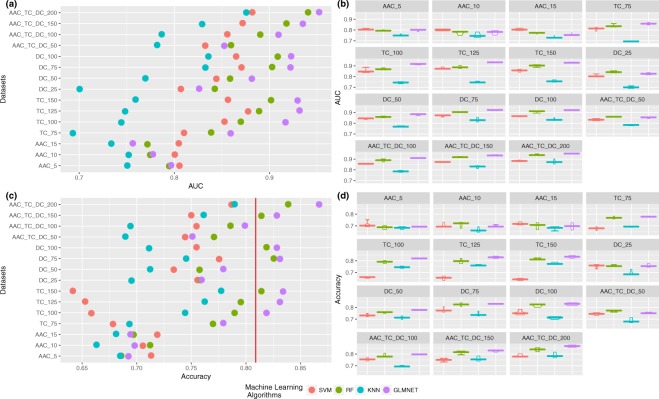
Results obtained in the feature selection process. (**a**) Summary of the performance of the four algorithms (AUC), (**b**) boxplot of the behavior of each model across experiments (AUC), (**c**) summary of the performance of the four algorithms (accuracy), and (**d**) boxplot of the behavior of each model across experiments (accuracy). The red line represents the best previously published value in the literature by Ramaprasad *et al*.^8^.

The results for each model obtained after feature selection are shown in Fig. 4. Therefore, at this point, the feature selection process shows the most relevant features–in this case, the amino acid residues and sequences of two and three amino acids–that better discriminate between the group of anti-angiogenic peptides and non-anti-angiogenic peptides. These results may have a great impact on later wet studies. This screening process hopefully minimizes the search for important sequences in anti-angiogenic peptides.

**Figure 5:**
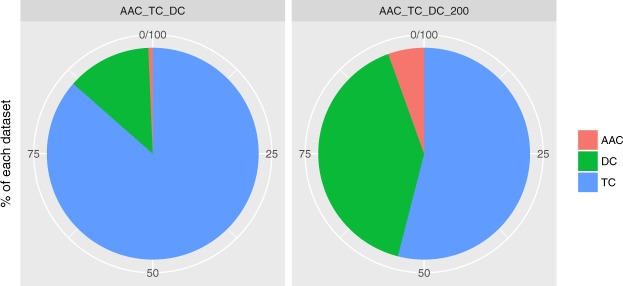
Percentage of the variables of each descriptor in the best-performing dataset before (3058 variables) and after (200 variables) the feature selection approach. An increase in the relative quantity of the AAC and DC descriptors is observed.

**Figure 6:**
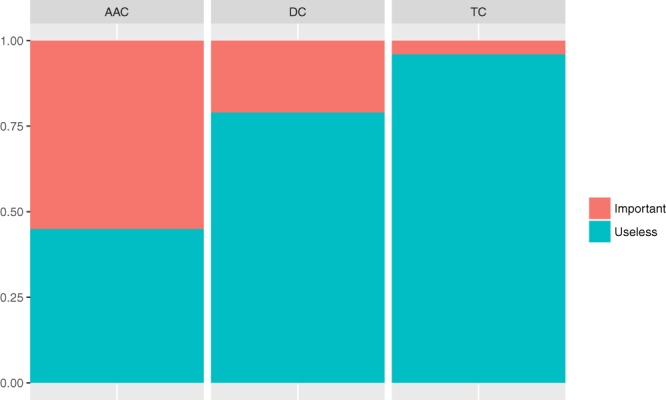
Relative proportion of the discarded variables (in blue) of the descriptor after applying the FS approach in the best-performing dataset.

The best model in terms of both AUC (see Fig. 4a) and accuracy (see Fig. 4c) has been the glmnet algorithm. The algorithm was trained with the union of the three datasets (AAC, DC and TC) and only the 200 features with the highest rank. In this context, it seems that the glmnet and RF algorithms work better than the others after the feature selection process. In addition, the results seem to indicate a dramatic improvement in the behavior of the algorithms, as seen in Fig. 4b,c. All models show a low variance in their results across experiments. Furthermore, in Fig. 5, the percentage of features of each of the datasets in the combination is shown. An increase in the importance of the features from AAC and DC is observed along with a decrease in TC. Remarkably, the percentage of important features (best model) versus useless features in the datasets is shown in Fig. 6.

Moreover, after the feature selection process, a significant improvement in the performance of the models was obtained, with an average of approximately 15% in accuracy and AUC for all models. The feature selection process works to reduce the noisy features.

The red line in Fig. 4c indicates the best result from the literature for anti-angiogenic peptides by Ramaprasad *et al*.^8^ (*accuracy* = 0.809). We statistically outperformed the literature with our experiments and experimental design with more than ten combinations of algorithms and descriptors.

### Best model determination

The final step in our experimental design^34^ is the statistical significance comparison of the performance (AUC) of the machine learning models. As shown in Fig. 4c, seven models outperform the state-of-the-art approach. We used these models to evaluate the statistical significance. Parametric tests have more power than non-parametric tests but, unfortunately, can be used only under certain circumstances. Thus, we checked the normality with a Shapiro-Wilk test, with a level of confidence *α* = 0.05 and the null hypothesis that the data follow a normal distribution; this was rejected with values W = 0.9302 and *p*-*value* = 0.02836. We performed a Bartlett test with the null hypothesis that our results are heteroscedastic, and we could not reject the null hypothesis with a value for Barlett’s K squared of 3.2445 with 6 degrees of freedom and *p*-*value* = 0.7776. In this case, one of the conditions does not hold, so following the tests, we performed a non-parametric Friedman test with the Iman-Davenport extension assuming the null hypothesis that all models have the same performance; this was rejected with *p*-*value* = 1.8665 × 10^−9^. A Finner post hoc procedure must be used to correct and adjust p-values for multiple comparisons. Hence, after this test and multiple comparison corrections, the null hypothesis was rejected for all models except the best models using datasets TC_125 and AAC_TC_DC_150, which performed statistically equally to the winning glmnet model with dataset AAC_TC_DC_200.

## Discussion

**Figure 7:**
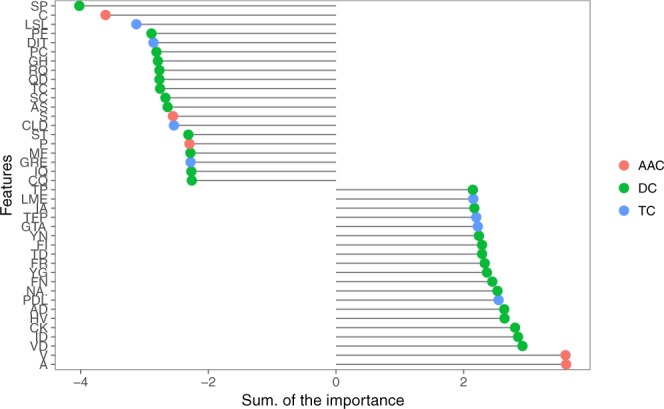
Variable importance of 200 features of the glmnet algorithm.

The top-40 features of the winning model are shown, for clarity reasons, in Fig. 7. We add the beta value (importance) of the glmnet model for each fold and experiment to understand the global importance of each feature. We plotted in different colors to show features belonging to a particular original dataset to clarify the feature selection process. We mentioned before in Fig. 2a that the use of AAC, TC or DC features alone or in groups of two is not enough because the datasets, in general, are noisy. In fact, our results show that a feature selection process with a combination of them can outperform our previous results and, more importantly, those in the literature. Furthermore, the best individual subgroup results were found with the AAC dataset followed by DC and TC, as shown in Fig. 2a. The discriminatory power found in the feature selection process is shown in Fig. 7, where the combination of the most informative features from different datasets, mostly from DC, allowed us to increase the knowledge about peptides.

The variables shown in Fig. 7 represent the proportion of residues, the sequences formed by two and three amino acids.

The three residues with a negative sum of betas obtained in our model (C, S and P) have also been reported by Ramaprasad *et al*.^8^. Furthermore, Karagiannis *et al*.^9^ reported that the CXC domain is prevalent in anti-angiogenic activity, which supports the presence of C residues in our model. However, it has been reported that residues such as Val and Ala are prevalent in non-anti-angiogenic peptides^8^, and in this study, these two residues obtain the major score of betas in this peptide activity.

In addition, the model has associated the presence of sequences formed by two or three amino acids with great power of discrimination for anti-angiogenic activity. In previous studies, the analysis of motifs in anti-angiogenic peptides has shown that motifs such as CG-G, TC, SC, SP-S, W-S-C, WS-C are most predominant in this type of peptide^8^. In our model, sequences such as SP, TC and SC also have great importance. In addition, the work reported by Vazquez RodrÃguezÂguez, G. *et al*.^35^ shows a list of peptide sequences with vasoinhibins activity. After a thorough analysis of their sequences, it has been observed that there is a high prevalence of sequences such as SP, SC, MF, IQ, CQ and HG, all of which have been reported in this work.

Corresponding to sequences formed by three amino acids, LSL and GRE sequences have been found in the Anginex peptide, an artificial peptide with high anti-angiogenic activity^36^. Although these sequences do not belong to the functional part of the protein, as Dings *et al*.^28^ reported, they have a role in the creation of bonds that generate and stabilize the secondary structure of the protein. In addition, LSL sequences have great prevalence in aminoacidic sequences of vasoinhibins, as reported by Vazquez Rodríguezguez, G. *et al*.^35^.

From a review of the state of the art, it seems that the top 20 variables with a negative sum of betas are related to anti-angiogenic activity, while a positive sum of betas is related to non-anti-angiogenic activity. Therefore, sequences with a positive sum of betas, such as PF, DIT, PC, GH, RQ, QD, AS, CLD and ST, which have been reported in this work but without any reference in the literature, can generate new knowledge on amino acid composition in anti-angiogenic activity.

The information and knowledge derived from this study are not based on any biological assumption. This study has therefore generated a new approach regarding the amino acid composition of anti-angiogenic peptides. This is a poorly known factor to which few scientific studies have paid attention. In addition, this work adds several levels of complexity in the study of this matter. First is the presence of molecular descriptors that refer to sequences formed by three amino acids, which has never been reported. Second is the union of variables from different descriptors, increasing the molecular information in a unique dataset. Finally, the implementation of FS approaches has significantly helped improve the performance of the models, exceeding on several occasions the model reported by Ramaprasad *et al*.^8^, which has been the one with the highest accuracy.

## Conclusions

This paper presents classification models of anti-angiogenic peptides with the best performance reported to date using four different machine learning models and molecular descriptors obtained through the Rcpi package of Bioconductor.

The results obtained in this work support those obtained in the literature related to anti-angiogenic activity such as Ramaprasad *et al*.^8^. After analyzing the importance of the variables, the model considers that the presence of the amino acid sequences SP, LSL, PF, DIT, PC, GH, RQ, QD, TC, SC, AS, CLD, ST, MF, GRE, IQ, CQ and HG is critical to distinguish where a peptide exhibits anti-angiogenic activity.

Because the model shown in this article did not work at any time under biological assumptions, it provides a more comprehensive approach than biological studies that failed to decipher their results. This is why further studies are needed to demonstrate the existing biological relationship between the variables most related to the anti-angiogenic activity presented in this work and their possible biological interactions. In addition to this approach, more *in silico* studies are necessary that examine the interaction between these peptides and the target proteins of our organism.

Since user-friendly and publicly accessible web servers represent the future direction for practically developing more useful models^37–40^, we shall endeavor in our future work to provide a web server for the method presented in this paper.

## Materials and Methods

### Dataset

The data were obtained from Ramaprasad *et al*.^8^. This dataset represents a list of peptide sequences classified according to their activity into two classes: anti-angiogenic and non-anti-angiogenic. The number of sequences in each class is 107. None of the peptides have an identity equal to or greater than 70% with any other of the positive peptides. The understanding of the biological process of angiogenesis is critical to understand how malignant tumors are formed in the body. The peptides were collected from various research articles and patents (10.1371/journal.pone.0136990.s007). As there is no source of experimentally proven non-anti-angiogenic peptides, the authors extracted a similar number of random peptide regions from proteins from the Swiss-Prot database^41^ and treated them as non-anti-angiogenic peptides (10.1371/journal.pone.0136990.s003). Though some of these randomly selected peptides could be anti-angiogenic in nature, the probability is very low.

The dataset consists of 107 peptide sequences classified as anti-angiogenic and 107 as non-anti-angiogenic. Following an initial check to ensure that all sequences presented a correct nomenclature, two of them were found to be erroneous and were eliminated from the database. The study therefore consisted of a total of 107 anti-angiogenic and 105 non-anti-angiogenic sequences. This type of balanced data is most suitable for use as inputs to the algorithms, as we ensure that there is no probabilistic tendency to classify a peptide in a specific class. The set of sequences were converted into three types of physicochemical descriptors (see the materials and methods) based on the primary sequence of the peptides. This way, we converted these sequences into a mathematical description of the aminoacidic composition of each peptide. After removing the variables of each descriptor that presented zero value in all observations, 2645 variables were obtained for TC, 20 variables for AAC and 393 variables for DC.

To conduct this study, amino acid sequences of peptides classified according to anti-angiogenic or non-anti-angiogenic activity were used. These sequences were converted into molecular descriptors, from the Rcpi^42^ package present in the Bioconductor project^43^. Subsequently, the datasets were subjected to multivariate analysis and classification methods based on machine learning algorithms to determine the model that presents the best performance in the classification of these peptides.

### Obtaining molecular descriptors

For the comparison and classification of peptides according to their activity, additional information from their sequence must be extracted. The package Rcpi^42^, presented in the Bioconductor project^43^, offers the possibility of obtaining structural and physicochemical characteristics of peptides from their amino acid sequences. In addition, it is highly interesting to gather information on characteristics as heterogeneously as possible to try to best determine all molecular information. Thus, machine learning algorithms, underlying knowledge from the data, will be able to obtain information covering as much solution space as possible.

In this study, we have worked with three types of molecular descriptors that have been calculated from different amino acid sequences: amino acid composition (AAC), dipeptide composition (DC) and tripeptide composition (TC)^44^. These descriptors are characterized by describing the composition of the amino acid sequence by easily interpretable variables. Below is a brief description of each set of descriptors.

#### AAC

This descriptor calculates the composition of each amino acid within the sequence. It reports an output with a total of 20 features/dimensions, each corresponding to an amino acid. The composition of each amino acid is obtained with the fraction of each type of amino acid within the peptide sequence^44^. Thus, Equation 1 is used for calculating the fraction of the 20 natural amino acids:

1 $${\rm{Fraction}}\,{\rm{of}}\,{\rm{aai}}=\frac{{\rm{total}}\,{\rm{number}}\,{\rm{of}}\,{\rm{amino}}\,{\rm{acids}}\,{\rm{of}}\,{\rm{type}}\,{\rm{i}}}{{\rm{total}}\,{\rm{number}}\,{\rm{of}}\,{\rm{amino}}\,{\rm{acids}}\,{\rm{in}}\,{\rm{protein}}}$$

where i is a specific type of amino acid.

#### DC

This type of protein descriptor calculates the percentage present in each sequence of all possible combinations of the 20 amino acids pairs. Because there exist in nature 20 different amino acids, there exist 400 possible pairs (20^2^). Therefore, a count is made of the pairs of adjacent amino acids found in each sequence. We adopted the same dipeptide composition-based approach as^44^, which involves calculation of the following equation, as a fraction of amino acids considering their local order (Equation 2):

2 $${\rm{Fraction}}\,{\rm{of}}\,{\rm{DC}}({\rm{i}})=\frac{{\rm{total}}\,{\rm{number}}\,{\rm{of}}\,{\rm{DC}}({\rm{i}})}{{\rm{total}}\,{\rm{number}}\,{\rm{of}}\,{\rm{all}}\,{\rm{possible}}\,{\rm{dipeptides}}}$$

where DC(i) is one dipeptide i of the 400 possible dipeptides.

#### TC

similar to DC, TC calculates the percentage of all possible tripeptides that can be found in a sequence. The tripeptide composition was used to transform the variable length of proteins to fixed-length feature vectors. The tripeptide composition gave a fixed pattern with length equal to ${20}^{3}$.

### Machine learning

Machine learning is the field of study interested in the development of computational algorithms capable of transforming data into intelligent actions. This field is extensive in several areas, as it helps explain and extract specific knowledge from a set of data that humans would not be able to achieve. The algorithms used are designed to perform a probabilistic search working in large spaces that involve states that can be represented by datasets. There are two main types of learning: supervised and unsupervised. The main difference between them is that in the former, learning occurs via labeled observations, while in the latter, the examples are not labeled, and the algorithm seeks to cluster the data into different groups. In this study, we will work with supervised classification algorithms from a set of labeled examples; these algorithms try to assign a label to a second set of examples.

We used four different implementations of the following machine learning algorithms: random forest (RF)^45^, K-nearest neighbors (k-NN)^46^, support vector machine (SVM)^47^ and a generalized linear model (glmnet)^48^.

Each of these machine learning algorithms has a particular set of hyperparameters that should be tuned to find the best possible combination and, consequently, the best prediction of and solution to the problem. Machine learning algorithms are very powerful techniques, but the training process is critical. This kind of algorithm learns through samples, so the same samples should not be used for learning, validation or hyperparameter tuning. We explain in further detail our robust experimental design in the experimental design section.

Random forest (RF) was developed by Breiman^45^ and consists of an ensemble of independent decision trees based on random resampling of the variables for the construction of each tree. A majority vote of the trees in classification is taken as the prediction. Thus, RF adds an additional layer of randomness to a conventional bagging approach.

A search was made of the appropriate values for the parameters mtry (number of variables randomly sampled in each division of the data) and nodesize (minimal size of the terminal nodes). The range for the number of variables was established between 1 and, as the upper limit, the square root of the number of variables with the largest dataset. The minimal size of the terminal nodes ranged between 1 and 3. Low values for this parameter provide great growth and depth of each tree, improving the accuracy of predictions. In addition, the number of trees was 1000. A large number of trees ensures that each observation is predicted at least several times.

The K-nearest neighbor (k-NN) algorithm is a technique based on cluster theory. It is a very basic algorithm, but it has been reported to yield excellent results for classification. In this case, we used a variant called weighted k-NN^49^. It is based on the fact that a new observation that is particularly close to an observation within the learning set should have a great weight in the decision and, conversely, an observation that is at a farther distance will have a much smaller weight^50^. The observations are mapped following the Minkowski distance.

For this algorithm, only the hyperparameter k has been tuned, which represents the number of neighbor data points that are considered closest. Because a very high k can cause over-training of the model, the decision was made to maintain intermediate levels. The range of values used was from 1 to 5.

The objective of support vector machines (SVMs) in binary classification problems is to obtain the best hyperplane that separates the two classes, thus minimizing the error. The hyperplane is defined through support vectors. Since most real problems do not have a linear relationship, the SVM algorithm offers the possibility of calculating a kernel function to map the data in a greater number of dimensions, making it possible to linearly separate the data^51^. There are different kernel functions. For this study, the kernel function RBF (Gaussian radial basis) was used.

The values for the hyperparameters C and sigma were searched, both with a range between 2^−12^ and 2^12^ with a step size of one–i.e., 2^−12^, 2^−11^, 2^−10^…. The modification of C implies an adjustment of the penalty of the misclassified observations. Sigma represents the standard deviation of the Gaussian distribution.

Logistic regressions are popular classification algorithms in machine learning problems when the response variable is categorical. The logistic regression algorithm represents the class-conditional probabilities through a linear function of the predictors. In this study, we use a fast regularization algorithm that fits a generalized linear model with elastic-net penalties, called glmnet. The algorithm was developed by Tibshirani *et al*.^48^. The elastic-net penalty can tend towards the lasso penalty^52^ to the ridge penalty^53^. The ridge penalty is known to shrink the coefficients of correlated predictors towards each other, while the lasso tends to pick one of them and discard the others. Therefore, the elastic-net penalty mixes these two.

The grids of alpha and lambda for tuning are (0.0001, 0.001, 0.01, 0.1, 1) and (0, 0.15, 0.25, 0.35, 0.5, 0.65, 0.75, 0.85, 1), respectively. Alpha controls the elastic-net penalty, from lasso (*α* = 1) to ridge (*α* = 0). The lambda parameter controls the total force of the penalty.

### Feature selection

The number of high-dimensional datasets is skyrocketing, and some of the features are redundant or noisy. To better explore the space of solutions, the number of useless features should be reduced as much as possible. The ultimate goal of the FS approaches is to find a subset of features from the original that contains as much information as possible without altering the original representation of the data. Furthermore, this subset should increase or at least not decrease the performance of the models. At the same time, it should prevent over-fitting and allow the fastest generation of better models^54^. Therefore, the redundant, noisy variables^12,54,55^ are eliminated along with, generally, the variables that are more correlated without providing new information.

In machine learning, there are three main approaches for FS, known as filter, wrapper and embedded^55^. The main difference between the filter approach and the other two is that the filter approach searches for the features selected independently of the classification algorithm, while the wrapped and embedded approaches search for the feature selection depending on the classification algorithm.

Filter approaches obtain a score that measures the relevance of the features against the class vector by observing only the intrinsic properties of the data without taking any assumptions from the classifiers. In addition, this approach is computationally simple and fast. It is especially relevant for high-dimensional data. As these approaches are independent from the classification algorithm, the subset of selected features is used as the input to any algorithm. There are two different filter approaches: univariate and multivariate. Univariate filter approaches are fast, scalable and independent of the classifier but ignore feature dependencies and interaction with the classifier. Multivariate models feature dependencies with independence of the classifier and are thus computationally better than wrapper methods. The reasons for using filter feature selection (univariate) in peptide prediction are its easy-to-understand output feature ranking, higher speed than multivariate approaches and ease of validation by biological lab methods and the fact that experts usually do not need to consider descriptor interactions^55–57^. Therefore, this approach allows us to perform a better comparison among the different classification models^55^ and particularity of each feature, independently of the particular behavior of each technique.

Therefore, we followed a univariate filter FS approach, and for the calculation of the relevance of the variables, a T-test was used. The T-test is one of the most robust parametric univariate statistical tests and one of the most widely used in the literature. Several sizes of features have been extracted from each of the three sets of descriptors under study—in a growing approximation, the minimal number of features most suitable to solving the problem.

### Experimental design

The experimental design of this work is based on the classification of peptides into two different classes: anti-angiogenic and non-anti-angiogenic. The dataset consists of primary amino acid sequences of two classes of peptides, represented by the nomenclature of a letter (A, R, N, etc.).

We used the Rcpi^42^ package from the Bioconductor project^43^ to calculate different descriptors for each sequence (AAC, DC and TC). In addition, the set of descriptors was merged to obtain the subgroup of descriptive variables coming from each descriptor with a greater prediction capability for the peptide activity, which was saved in a unique database. Finally, the data were standardized so that the distribution of the sample has an average equal to zero and a standard deviation equal to one. The output obtained from the FS approach was used in the training and evaluation of the different classification algorithms.

A nested resampling was used for the training of the models. The characteristic of this process is the presence of an independent internal cross-validation (2/3 for training and 1/3 for validation) for the selection of the best hyperparameters of each algorithm and an independent external cross-validation (5 repetitions of a 10-fold-CV) to evaluate the model in a general way. For each 10-fold-CV experiment, the peptide sequences were randomly divided into ten sets. Nine sets were used for training the model, and the remaining set was used for testing. The process was then repeated ten times such that each set was used once as a test set. The average performance of all ten sets was reported as the final performance of the method. We repeated this process 5 times for each ML algorithm, and we presented the mean average of the 5 runs in the figures of the paper.

The performance of the different experiments was determined through the package mlr^58^. This package facilitates the design of machine-learning-based experiments, reducing the amount of scripting needed and providing a simpler and more manageable platform for development while facilitating reproducibility and replicability. Moreover, this package ensures that the execution of the machine learning algorithms follows the experimental design under the same conditions, thus allowing the comparison under equality of conditions. For the evaluation of the models, we used accuracy (to compare our findings with the state of the art) and the area under the ROC (AUC) to control for type I and II errors.

Finally, the finding of the best results and the analysis of the statistical significance of the results were carried out by means of null hypothesis tests. Furthermore, the importance of each particular descriptor in the best final model was analyzed and compared to previous findings in the literature.

## Data Availability

The R script code for dataset generation is available for download at 10.6084/m9.figshare.6016994.
